# Research and Remedies

**Published:** 1911-12-16

**Authors:** 


### RESEARCH AND REMEDIES.

### THE ABUSE OF SILVER SALTS.

Within the last seven or eight years various preparations of silver—most of them organic compounds of the metal—have been taken very largely into favour by ophthalmologists and others who were formerly accustomed to use solutions of silver nitrate. The most widely used and accepted of these organic preparations are probably argyrol and protargol, though there are many others which are also much prescribed. Theobald, Professor of Ophthalmology at the Johns Hopkins University, protests very emphatically against the indiscriminate use of these preparations, on the ground that cases of ocular argyria are by no means uncommon after their use, though such a complication is extraordinarily rare when the nitrate is ordered. As to the efficacy of the organic compounds of silver he is quite clear, nor does he wish to protest against their use altogether; for he relies on them exclusively in the treatment of gonorrhœal conjunctivitis, both in the infant and the adult, and of trachomatous conjunctivitis. Rather it is the routine and almost universal habit that he objects to, into which the younger generation of ophthalmologists and general practitioners have fallen, of using protargol or argyrol in the treatment of acute and chronic catarrhal conjunctivitis. This practice is, he says, indefensible, for, a collyrium containing half a grain of zinc sulphate and ten or twelve grains of boric acid to the ounce is cleaner, is not liable to stain the conjunctiva, and is more surely and more promptly efficacious in these conditions than any of the silver compounds.

### CAUSE AND TREATMENT OF CANCER.

Two publications of the past month on this elusive problem are worthy of mention. The first is Sir Henry Butlin's theory of what cancer is, outlined in two communications to the *Lancet*. Put briefly, he regards the cancer cell as a definite organism, which he terms "unicellula cancri"; and for it he is prepared, if necessary, to establish a place in zoology as a separate order of animal not included in any division or sub-division of our present classifications. He answers the riddle of the supposed cancer parasite by asserting that the cancer cell *is* the parasite; and that a cancerous growth, therefore, which is an accumulation composed largely of cancer cells, is a colony of cancer parasites. It is evident that this bold generalisation solves several of the difficulties (that of metastasis, for instance) which the natural history of cancer has long presented; but it is premature at present to decide what value is to be placed on this hypothesis. Sir Henry's view is far from being proved to be scientifically tenable, but, at least, it is bound to direct research and thought into new channels. More than that at present it is scarcely possible to say.

The other publication is a volume in which Dr. John Beard summarises his present position in regard to the nature and treatment of cancer.* This author labours under a great grievance; he believes he has discovered the nature of cancer and how to cure it, but he cannot get the medical profession to admit his claims. By far the greater part of his book is taken up by vehement protests against the conspiracy of silence with which he believes his theories have been unjustly pooh-poohed. Page after page is filled with rambling diatribes against British scientists of repute; and Dr. Beard does not hesitate to compare himself, as an unrecognised discoverer, with Galileo, Pasteur, Semmelweis, and other celebrated men. In a word, the dominant note throughout is one of egotism, and of particularly arrogant egotism at that. This should not, of course, prevent a careful examination of the arguments and results which the author adduces in support of his views; though naturally it does not predispose anyone in his favour. Dr. Beard will have it that his methods of treating cancer with trypsin and amylopsin have not had a fair trial; and especially that the failures of methods which he has now abandoned are quoted against him. Yet his claims for these abandoned methods were just as large a few years ago as his claims for his present methods are now; and he really cannot complain when those who tried his older "cure for cancer" and found it wanting, remain a little shy of testing a similar method which has now superseded the other in his affections.

At the same time it is interesting and notable that an officer of the Royal Army Medical Corps has had success in treating cases of apparently undoubted cancer by Dr. Beard's ferment methods. The case of which photographs and microphotographs are reproduced does seem to stand the test of examination; and though it is early yet to decide that a complete and permanent cure has been secured, at least it may be freely granted that the progress of the case has been remarkable. Assuming, then, for the sake of argument that this case is a cure, and that the ferment treatment produced the cure, Dr. Beard has still to show that his method produces results which in bulk are superior to those of surgery. He ought to show by full statistical tables the percentage of his cases in which cure has taken place, and to prove that operative treatment fails to produce as good a result. Instead of this he commits himself to the ridiculous statement that it is a "scientific absurdity" to assert that surgery "ever knowingly cures a single case of cancer." The truth is, in a nutshell, that surgery cures a proportion of cancer cases (and by "cure" is meant a true permanent cure); that this proportion has been increasing steadily for many years; and that Dr. Beard's method has been tried far and wide in Britain and abroad with such uniform lack of success that it is unjustifiable to continue it further.

* *The Enzyme Treatment of Cancer and its Scientific Basis.* By John Beard, D.Sc. London: Chatto and Windus. 7s. 6d. net.

